# Telomere length from peripheral blood DNA in major depressive disorder: a case/control comparison and association with antidepressant treatment response

**DOI:** 10.1192/j.eurpsy.2023.1259

**Published:** 2023-07-19

**Authors:** R. Kalinovic, C. Homorogan, D. Nitusca, E. Seclaman, C. Marian, B. Adela, V. R. Enatescu

**Affiliations:** 1 Psychiatry; 2 UMFT, Timisoara, Romania

## Abstract

**Introduction:**

Major depressive disorder (MDD)is one of the most common psychiatric disorders and a large number of patients present a poor response to treatment. Shortening of telomeres physiologically occurs after each cell division and their shortening is also associated with cell ageing. Studies are affirming that patients who suffer from mental illnesses have shorter telomeres in comparison with patients without such affections. ^4^ The effect of antidepressant medication on the biology of the telomeres was studied very little in humans and the current data suggest that the length of telomeres is a promising target regarding the prognosis and the response to psychotropic treatment.

**Objectives:**

To analyze the telomere length (TL) from peripheral blood DNA of patients with major depressive disorder (MDD) compared to healthy controls. A second objective was to compare the TL of patients in relation to antidepressant treatment.

**Methods:**

We analysed the clinical data from 16 patients admitted to the Psychiatry Clinic of Timisoara with the diagnosis of MDD and 10 healthy controls. The collection of clinical data was carried out in a structured and standardized manner both on paper and electronically, and the Hamilton Depression Rating Scale (HDRS) was applied to assess the severity of depression. Also, blood samples were collected, and plasma and white blood cells (WBC) were separated by centrifugation. Patients’ samples were collected before and after 12 weeks of escitalopram antidepressant treatment, and a structured diagnostic interview and a standardized depression rating scale were done. DNA was extracted from WBC using the QIAamp DNA Mini Kit (Qiagen), and TL was determined by real time PCR using the Absolute Human Telomere Length Quantification qPCR Assay Kit (Sciencell Research Laboratories) according to the manufacturers’ specifications. The TL expressed as megabases/diploid cell (Mb/DC) were compared between cases and controls using a Mann-Whitney test, and between patients before and after treatment using a Wilcoxon matched-pairs signed rank statistical test.

**Results:**

The mean±SD telomere length for healthy controls was 0.3614±0.082Mb/DC, for treatment naïve patients was 0.4513±0.199Mb/DC, and for patients after treatment was 0.3476±0.070Mb/DC. There was no statistical significant difference in TL between patients and controls (p=0.266), nor between patients before and after treatment (p=0.055).

**Conclusions:**

In this pilot project of limited sample size there was no difference in TL between MDD patients and healthy controls. Moreover, the TL of patients did not significantly change after 12 weeks of escitalopram antidepressant treatment.

**Disclosure of Interest:**

None Declared

